# Tetrastarch in cardiac surgery: error, confounding and bias in a meta-analysis of randomized trials

**DOI:** 10.1186/s13054-015-0907-8

**Published:** 2015-04-22

**Authors:** Roberta J Navickis, Gary R Haynes, Mahlon M Wilkes

**Affiliations:** Hygeia Associates, 17988 Brewer Road, Grass Valley, CA 95949 USA; Department of Anesthesia Services, Cabell Huntington Hospital, 1340 Hal Greer Boulevard, Huntington, WV 25701 USA

In a meta-analysis of cardiac surgery trials, we showed that hydroxyethyl starch increases postoperative blood loss, blood product transfusion and reoperation for bleeding [1]. Citing that meta-analysis, the US Food and Drug Administration determined excess bleeding to be a class effect of hydroxyethyl starch solutions and issued a safety warning [2].

**Figure 1:**
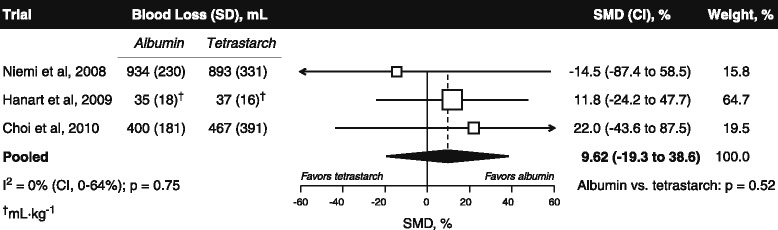
**Partially corrected meta-analysis of postoperative blood loss in the three trials included by Jacob and colleagues comparing tetrastarch with albumin [** 3 **].** Upon request, individual patient postoperative blood loss data were supplied by Niemi and colleagues [6] and the means and standard deviations of cumulative 24-hour postoperative blood loss by Choi and colleagues [7]*.* The data provided by Niemi and colleagues reveal that the true blood loss difference in their trial was 41 ml, not 95 ml as imputed by Jacob and colleagues*.* Both groups in the trial by Hanart and colleagues received albumin postoperatively [8], while Choi and colleagues infused tetrastarch in both groups postoperatively. Reliable correction for confounding in those two trials is not feasible, so these data are subject to bias in favor of tetrastarch. Test fluid administration was limited to the postoperative period in the trial of Niemi and colleagues*.* Data combined under a fixed-effects model. Error bars indicate 95% confidence interval (CI). Data points scaled according to meta-analytic weight. SD, standard deviation; SMD, standardized mean difference.

Jacob and colleagues report a new meta-analysis suggesting lower perioperative blood loss with tetrastarch than albumin across three trials [3]. However, postoperative blood loss was in the opposite direction (Figure 1). By imputing key unreported data instead of contacting the trial investigators, Jacob and colleagues introduced major errors favoring tetrastarch; for example, inflating the blood loss difference in one trial by 2.3-fold. Furthermore, the other two trials were confounded by exposure of one group to both test fluids. The potential distortion is highlighted by a randomized trial in which coadministration of low-dose albumin with tetrastarch reduced blood loss by 21% (*P* < 0.05) versus tetrastarch alone [4]. Without confounding the blood loss differences would almost certainly have been larger, and any meta-analysis incorporating the confounded trials is likely to be biased in favor of tetrastarch.

Their finding of lower blood loss with tetrastarch than pentastarch is attributable to publication bias, since an unpublished trial with higher blood loss and more frequent reoperation for bleeding after tetrastarch was omitted [1,5]. The omitted trial had been submitted to the US Food and Drug Administration in a New Drug Application by the same tetrastarch manufacturer who commissioned the new meta-analysis. That trial was included in two previous meta-analyses [1,5].
